# Characterization of a modified ROCK2 protein that allows use of N6-ATP analogs for the identification of novel substrates

**DOI:** 10.1186/1472-6750-14-2

**Published:** 2014-01-09

**Authors:** Amber L Couzens, R Montgomery Gill, Michael P Scheid

**Affiliations:** 1 Department of Biology, York University, 4700 Keele Street, Toronto, ON M3J1P3, Canada; 2 Current Address: Centre for Systems Biology, Lunenfeld-Tanenbaum Research Institute, Mount Sinai Hospital, Toronto, ON M5G 1X5, Canada

**Keywords:** ROCK2, Protein kinase, Chemical engineering, Cell signaling

## Abstract

**Background:**

The Rho-associated coiled-coil kinase-2 (ROCK2) is an important signaling transducer in the transmission of extracellular signals effecting organization of the actin cytoskeleton. ROCK2 has been implicated in numerous pathologies and the current focus is on understanding the molecular events that couple ROCK2 activity to biological function. To aid in the search for new ROCK2 substrates, we have developed an analog-sensitive (AS) ROCK2 protein that allows the use of selective ATP analogs that are not efficiently utilized by other protein kinases.

**Results:**

The analog sensitive protein, M160A ROCK2, was highly active and could phosphorylate proteins from a cellular homogenate with ***γ***^32^P-N_6_ (benzyl)ATP. We show the utility of this approach by identifying a putative ROCK2 substrate, elongation initiation factor-1-α1. We further show that the major site of ROCK2 phosphorylation of EIF1α1 is Thr^432^.

**Conclusions:**

Our work demonstrates that AS-ROCK2 could be useful in a systematic proteomic approach for identifying novel ROCK2 substrates.

## Background

The Rho-associated coiled-coil kinase-2 (ROCK2) is a large serine/threonine kinase that plays diverse roles in the cell including contraction, motility and morphology (reviewed in [1]). In the absence of Rho-GTP, ROCK2, and its closely related homologue ROCK1, adopts an auto-inhibited form [2], and upon Rho-GTP binding auto-inhibition is relieved and the kinase adopts an open, active conformation [3]. Rho-GTP activates ROCK2 to alter the activity of the actin reorganization machinery. For example, ROCK2 activation indirectly increases myosin light chain (MLC) phosphorylation, through the phosphorylation and inactivation of MLC phosphatase (MLCP) [4]. ROCK2 can also directly phosphorylate MLC at Ser^19^[5], the same site targeted by MLC kinase. ROCK2 further causes the disruption of the head-to-tail association of ERM proteins, through the phosphorylation of ezrin, radixin and moesin [6]. In addition, LIM-kinase 2 is activated by ROCK2, which then phosphorylates its downstream target, cofilin [7]. Phosphorylation of cofilin inhibits its actin depolymerizing function, thus increasing the number of actin filaments and leads to reorganization of the cytoskeleton [8].

Many human cancers demonstrate increased ROCK2 activity, which can augment tumor invasiveness [9,10]. Animal models have revealed that ectopic ROCK2 activation in established tumors is sufficient to drive metastasis of tumor cells into the surrounding stroma [9]. ROCK2 has also been implicated in the pathogenesis of hypertension, since ROCKs play a crucial role in smooth muscle contraction [11], through phosphorylation of MLC and MLCP. Furthermore, ROCK2 has been shown to influence the expression of genes that are important in vascular function, such as plasminogen activator inhibitor-1 (PAI-1) [12] and osteopontin [13]. Since ROCK2 plays a role in a number of human diseases, this kinase has received considerable interest as a potential therapeutic target.

ROCK2 is a member of the AGC kinase family and shares homology within the catalytic domain with other AGC kinase members including PKA, PKB, PKC, S6K, and SGK. This has led to the realization that ROCK2, like other AGC kinases, could target sequences that fall within a characteristic phosphorylation motif of R/KXS/T or R/KXXS/T [14]. Substrate preference for ROCK2 phosphorylation of this consensus motif is likely governed by spatial and temporal constraints; for example PKA distinguishes *bona fide* substrates through a mechanism in which bridging between kinase and substrate is provided by adapter and scaffold molecules [15].

To identify new ROCK2 substrates, a potentially useful approach is to modify the kinase so that it utilizes an ATP molecule restricted from use by other kinases. For example, an ATP with a bulky hydrophobic group attached to the N_6_ position of the purine base prevents entry of the analog into the catalytic site of most protein kinases [16,17]. The kinase domain can be engineered to accept this analog by introducing a modification to sterically accommodate the ATP analog. This chemical engineering approach was first demonstrated in the prototypical protein tyrosine kinase v-Src [17] and has been adapted to various kinases leading to the discovery of many important new substrates [18,19].

Here, we have adapted this chemical engineering approach to ROCK2. We show that a modified ROCK2 harboring a single amino acid substitution in the catalytic domain resulted in a 100-fold decrease in the K_m_ for N_6_(Benzyl)ATP utilization compared with wildtype kinase. Phosphorylation of a cellular homogenate with γ^32^P-N_6_(Benzyl)ATP led to a mixture of highly phosphorylated proteins that were separated by 2D-gel electrophoresis. Mass spectral identification and biochemical analysis of one of these phosphorylated proteins, eukaryotic elongation factor-1-α-1 (eEF1α1), demonstrated the utility of this approach and provides an important reagent for the future identification of ROCK2 signaling targets.

## Results and discussion

### Generation of AS-ROCK2

**Figure 1:**
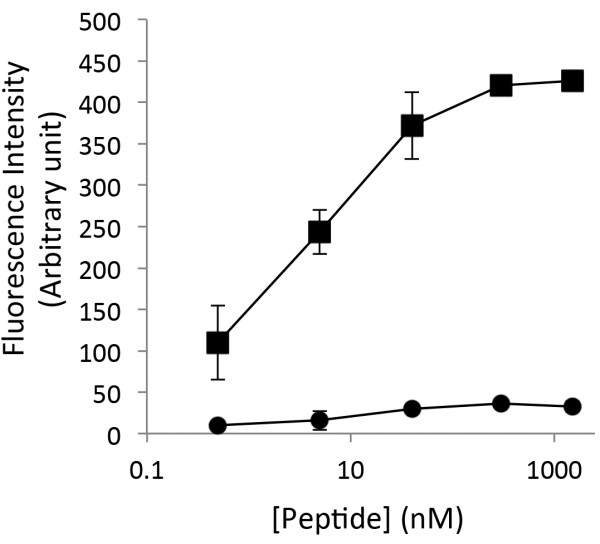
**Biotinylated LIMK peptide assay specificity.** Serial dilutions of Phospho-LIMK1 (DRKKRYpTVVGNPY; squares) and LIMK1 (DRKKRYTVVGNPY; circles) peptide with concentrations ranging from 4 μg/ml to 90 ng/ml were bound to streptavidin plates (Pierce) for 2 hours at room temperature (24°C). Wells were then washed and probed with phospho-LIMK (Cell Signaling) over night at room temperature. Secondary decoration with IRDye®680 anti-rabbit(Li-Cor) was performed at room temperature for 1.5 hours. Proteins were visualized by direct fluorescence scanning with a Li-Cor Odyssey Imager. Error bars represent the standard deviation of triplicate determinations and are representative of three independent experiments.

We were interested in identifying ATP binding-pocket mutations within ROCK2 that permitted the use of bulky ATP analogs. To screen for these mutations, we developed an *in vitro* nonradioactive assay based on phosphorylation of a biotinylated ROCK2 substrate peptide matching the consensus ROCK2 phosphorylation site in LIMK. Following phosphorylation *in vitro* by ROCK2, the biotinylated LIMK peptides were bound to a 96 well streptavidin-coated plate and phosphorylation was measured by probing with a commercially available phospho-specific antibody coupled to a secondary antibody conjugated to an 680 nm wavelength fluorochrome (IRDye680). Detection of the phospho-peptide antibody complex was performed directly on-plate using the Licor Odyssey infrared laser scanner. The assay was simple, rapid, and had a wide dynamic range comparing phospho-Thr^505^ LIMK fluorescence with the non-phosphorylated LIMK peptide (Figure 1). The unphosphorylated peptide was unreactive to the phosphospecific antibody up to the maximal concentration tested of 4 μg/ml.

**Figure 2:**
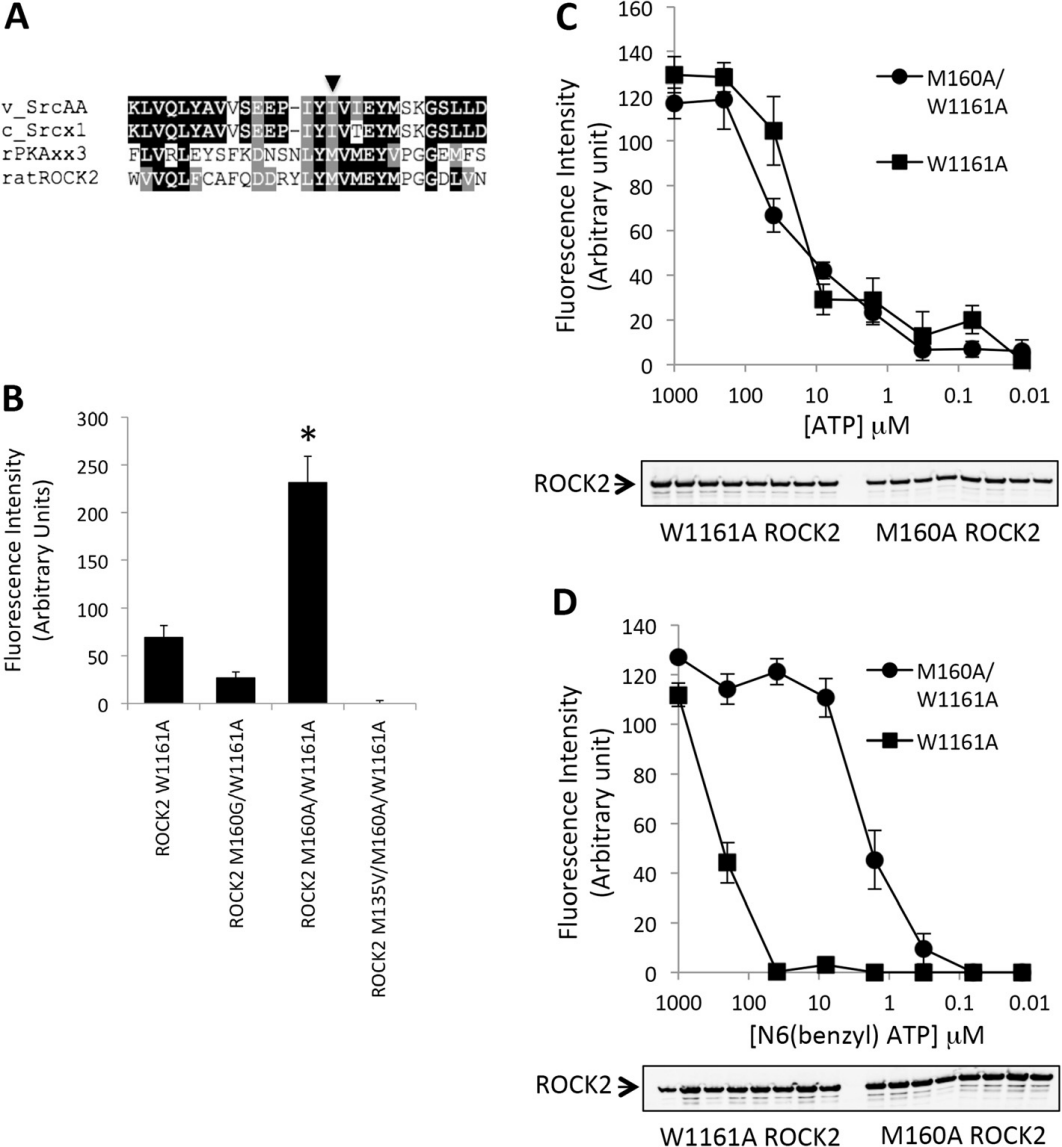
**Creation of AS-ROCK2. A**. ROCK2, V-Src, C-Src and rat PKA sequences were aligned using ClustalW. The v-Src ‘gatekeeper’ residue within the ATP-binding pocket is Ile^338^ (indicated by arrow) and is primarily responsible for restricting the ability of protein kinases from utilizing N6-substiuted ATP analogues. Ile^338^ in v-Src corresponds to Met^160^ in ROCK2. **B**. HEK 293 cells were transfected with various FLAG-ROCK2 constructs where indicated. The following day, cells were lysed and ROCK2 was immunoprecipitated with anti-FLAG conjugated agarose. LIMK peptide was phosphorylated using N_6_(Benzyl)ATP, loaded onto streptavidin plates (Pierce), and probed with P-LIMK (Cell Signaling) overnight at room temperature. Secondary decoration with IRDye® 680 anti-rabbit (Li-Cor) was performed at room temperature for 1.5 hours. LIMK peptide phosphorylation was visualized by direct fluorescence scanning with a Li-Cor Odyssey Imager. The relative LIMK phosphorylation signal is shown by the histogram for each ROCK2 mutation. Error bars represent standard deviation of triplicate determinations from three independent experiments. Asterisks indicate significant difference (P < 0.001) with W1161A ROCK2 as using Student’s *t*-test. **C**. Kinase activity of W1161A ROCK or W1161A/M160A ROCK2 was determined as in *B*, using serial dilutions of ATP with concentrations ranging from 1 mM to 12.8 nM. **D**. As in *C*, except N_6_(Benzyl)ATP was used. For *C* and *D*, error bars represent the range of duplicate determinations. Results are representative of three independent experiments.

For ROCK2, Met^160^ is analogous to Ile^338^ in v-Src (Figure 2A), where this single bulky residue of v-Src was shown to prevent the acceptance of N6-modified ATP analogues [20]. Mutation of the Met^160^ residue in ROCK2 to an alanine or glycine was modeled to yield the space required to accommodate N6(Benzyl)ATP. This mutation was introduced into the W1161A ROCK2 background as we have previously shown that this protein exhibits high kinase activity levels [21]. The Met^160^ to Ala substitution resulted in a 4-fold increase in substrate phosphorylation over wildtype ROCK2 at an N_6_(Benzy)ATP concentration of 100 μM (Figure 2B). The Met^160^ to Gly substitution had a 50% reaction velocity compared with the wildtype sequence, while Met^135^ to Val^135^ substitution also resulted in a major decrease in reaction velocity, indicating that these substitutions are inhibitory.

Since the Met^160^ mutation resulted in a greater velocity at 100 μM N_6_(Benzy)ATP than wildtype ROCK2, we analyzed this protein over a concentration range of analog to estimate K_m_ for N_6_(Benzyl)ATP. We saw no difference in the K_m_ concentration of ATP between wildtype ROCK2 and Ala^160^ ROCK2 (both ~30 μM, which is similar to the published K_m_ for ROCK2) (Figure 2C). In contrast, there was a 100-fold decrease in K_m_ for N_6_(Benzyl)ATP between the two kinases (~200 μM for wildtype ROCK2 and ~2 μM for Ala^160^ ROCK2) (Figure 2D).

### eEF1α1 is phosphorylated by AS-ROCK2 in vitro

**Figure 3:**
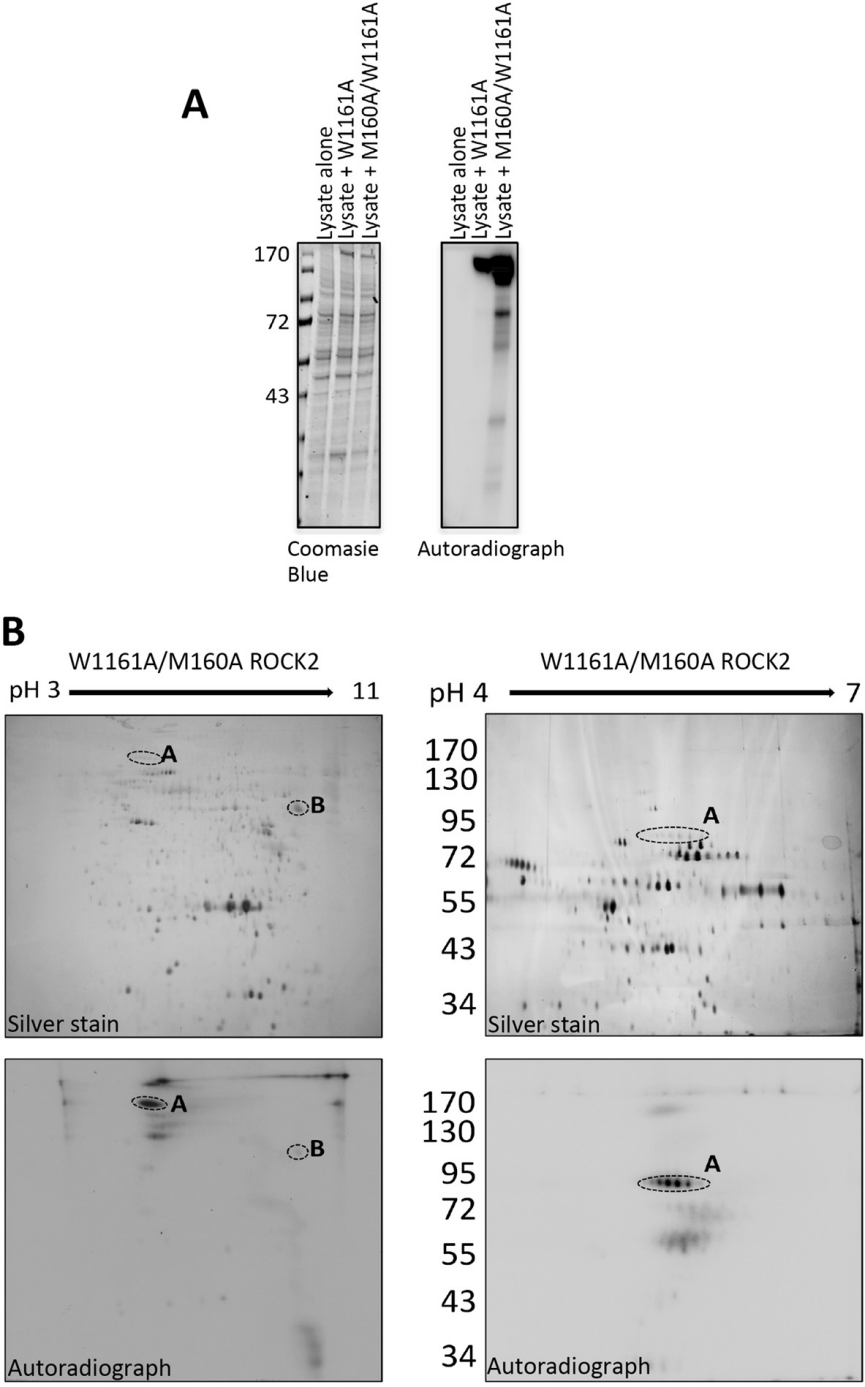
**Two-Dimensional separation of ROCK2-phosphorylated HEK293 cellular homogenate. A**. HEK 293 cells were transfected with either W1161A or M160A/W1161A ROCK2. The following day, ROCK2 was immunoprecipitated and resuspended in HEK 293 cellular homogenate (60 μg) with γ^32^P-N_6_(Benzyl)ATP in activation buffer for 30 minutes at 30°C. A small sample was taken and separated by SDS-PAGE followed by staining with Coomassie Blue and exposure to film. **B**. The remaining sample of M160A/W1161A ROCK2-phosphorylated homogenate was separated from the beads and proteins were precipitated with acetic acid/TCA, and then resuspended in 2D sample buffer. Proteins were loaded onto pH 3-11NL or pH 4-7 L IEF strips, as indicated. Following overnight IEF, the samples were separated in the 2^nd^ dimension by 8% SDS-PAGE. Gels were then silver-stained, dried (upper panel), and exposed to Amersham Hyperfilm (lower panel). Dashed circles indicate putative ROCK2 substrates that were excised and sent for identification. Results are representative of three independent experiments.

Next, we utilized M160A/W1161A ROCK2 to phosphorylate HEK293 cellular homogenate. Cellular homogenate was incubated with M160A/W1161A ROCK2 in the presence γ^32^P-N_6_(Benzyl)ATP (Figure 3A). In addition to autophosphorylated ROCK2, numerous other ^32^P-labeled proteins were clearly observed by autoradiography. The phosphorylated protein homogenate was then fractionated by 2D-electrophoresis and Figure 3B shows that at least eight proteins were phosphorylated by the exogenous M160A/W1161A ROCK2 protein. Two spots in particular were labeled strongly (*A* and *B*; Figure 3B), and one of them (*B*) was identified by mass spectroscopy to be the eukaryotic elongation initiation factor-1-α1 (eEF1α1).

**Figure 4:**
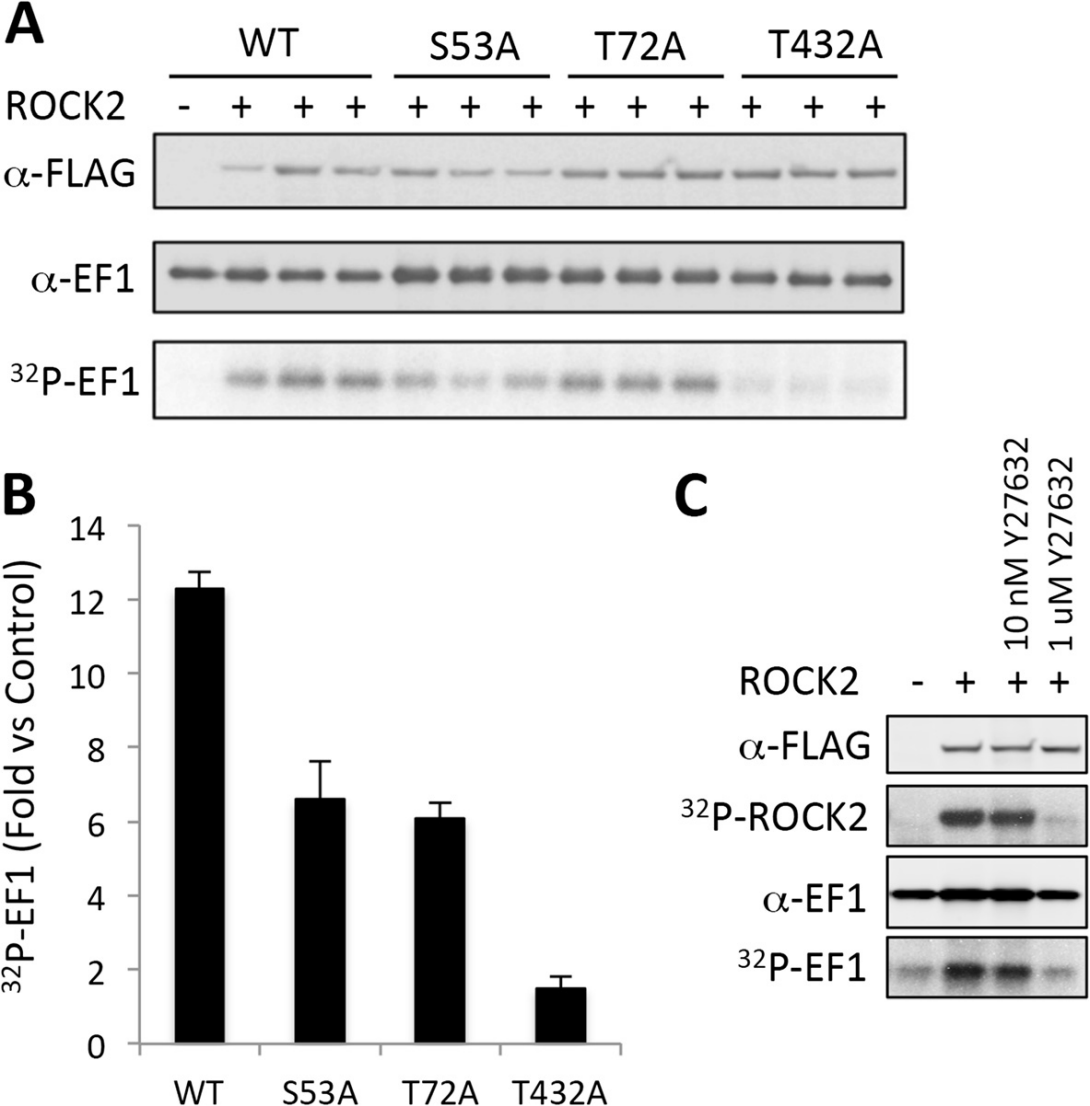
**Phosphorylation of EF1α1 *in vitro by *ROCK2. A**. EF1α1 (5 μg) was phosphorylated *in vitro* with W1161A ROCK2 as described in *Methods*. The samples were then separated by SDS-PAGE and transferred to a PVDF membrane. FLAG-ROCK2 was visualized by blotting with anti-FLAG M2 antibody, and EF1α1 was detected by anti-EF1α1 antibody. EF1α1 phosphorylation was visualized by phosphoimager. **B**. Fold increase in phosphorylation of EF1α1 by W1161A ROCK2 compared with no kinase for each of the Ser/Thr to alanine substitutions. Error bars represent the standard deviation of triplicate determinations from two independent experiments. **C**. EF1α1 was phosphorylated *in vitro* by W1161A ROCK2 in the presence of 10 nm or 1 μM Y27623. The figure is representative of two experiments.

We noted three sites of eEF1α1 (Ser^53^, Thr^72^, and Thr^432^) that fell within the ROCK consensus phosphorylation motif. These residues were exchanged for alanine and subjected to an *in vitro* kinase assay with W1161A ROCK2 (Figure 4). Thr^432^ substitution of eEF1α1 resulted in a 90% loss of phosphorylation, suggesting that Thr^432^ is a major site of phosphorylation by ROCK2.

The identification of new ROCK2 substrates is important for understanding how this essential regulator of cell mobility and contraction signals to control cellular events. Our study has provided a number of useful advancements in this regard. First, the biotinylated LIMK peptide assay was developed to quickly and quantitatively assess ROCK2 activity *in vitro* with phospho-specific antibodies and without radioactivity. The biotinylated LIMK peptide assay could be useful in future research applications that seek to assess ROCK2 catalytic activity *in vitro*, such as testing the catalytic effects of point mutations or the discovery of small compound inhibitors of ROCK2.

Secondly, we have utilized the biotinylated LIMK peptide assay to evaluate a ROCK2 mutation that allows utilization of N_6_(Benzyl)ATP. Since this bulky ATP analog is not efficiently used by ROCK2 (K_m_ of 200 μM; Figure 2D) or many other protein kinases [17,18], the M160A ROCK2 protein represents a valuable tool for the future identification of novel ROCK2 substrates. This modified protein was able to phosphorylate the LIMK peptide *in vitro*, and was able to phosphorylate an array of proteins in a cellular lysate that could be separated by two-dimensional electrophoresis and identification by mass spectroscopy. This resulted in the successful identification of the putative ROCK2 substrate eukaryotic elongation initiation factor-1α-1 (eEF1α1). The eEF1α1 is a highly conserved GTP-binding protein that interacts with aminoacyl-tRNA and recruits it to the ribosome during peptide elongation [22]. In addition to this role, eEF1α1 has also been found to be a part of a diverse number of cellular activities, including interactions with actin [23] mitotic apparatus complex formation [24], association with phosphorylated PKB [25], and interactions with PDK1 [26].

Izawa and colleagues reported that eEF1α phosphorylation inhibited co-sedimentation with F-actin [27]. eEF1α binds F-actin or aminoacyl-tRNA in a competitive manner [28]. Thus, eEF1α phosphorylation by ROCK may release the elongation factor from the cytoskeleton, allowing binding of aminoacyl-tRNA, leading to localized translation [27]. Other studies have since shown that overexpression of eEF1α2 leads to filopodia formation in human breast cancer cells, which was reversed with the ROCK2 inhibitor Y-27632 [29]. These results argue that further investigation is required to elucidate whether eEF1α1 represents a physiological ROCK2 substrate.

Examination of the eEF1α1 protein sequence revealed that residues Ser^53^, Thr^72^, and Thr^432^ all fall within a ROCK2 consensus motif R/KXS/T or R/KXXS/T (where X is any amino acid) [14]. Individual mutation of these residues to alanine followed by *in vitro* phosphorylation by ROCK2 showed that Thr^432^ is the major site of ROCK2 phosphorylation, since minimal phosphorylation was observed with T432A eEF1α1 (Figure 4). Future work will involve the generation of a phospho-specific antibody to Thr^432^ of eEF1α1 that will aid in elucidating the role of phosphorylation at this residue in cells.

Amano and colleagues [30] have previously reported an alternative proteomic approach to identify substrates of ROCK2, by combining mass spectrometry with affinity column chromatography. This method utilized the catalytic domain of ROCK2 as bait to probe a fraction of cytosol for interacting proteins [30], where our ATP analogue approach relies on a full-length protein that specifically phosphorylates substrates. Both techniques are similar in that proteins of interest were first separated from non-substrates, followed by identification by mass spectrometry.

Further identification of ROCK2 substrates using the ATP analogue approach will focus on specifically enriching for proteins phosphorylated by the AS-ROCK2 protein. This could be achieved by utilizing an N_6_-ATP-γ-S analogue and substrate enrichment with iodoacetyl-agarose resin, which binds the sulfonated group [31], prior to identification by mass spectrometry.

## Conclusion

In summary, our work has shown the utility of an AS-ROCK2 mutation that allows the use of bulky ATP analogs. This model will be of significant value for future efforts aimed at identifying novel ROCK2 substrates that could play a role in human disease such as cancer and hypertension.

## Methods

### Cell culture

HEK (human embryonic kidney)-293 cells were obtained from the A.T.C.C. (Manassas, VA, U.S.A.) and cultured in DMEM (Dulbecco’s modified Eagle’s medium) supplemented with 10% fetal bovine serum and antibiotics at 37°C in a humidified incubator containing 5% CO2.

### Reagents

Antibodies used were anti-FLAG M2 (Sigma-Aldrich), anti-[phosphoLIMK1(Thr^508^)/LIMK2(Thr^505^)] (Cell Signaling Technology) and anti-EF1α1 (H-300) (Santa Cruz Biotechnology).

### Site-directed mutagenesis

The M160A/W1161A and M160G/W1161A ROCK2 were generated using QuikChange® mutagenesis kit (Stratagene). Mutations were sequence-verified.

### cDNA transfections

HEK-293 cells were plated onto 100-mm dishes at 80% confluence and transfected with 2.5 mg of cDNA using Lipofectamine™2000 (Invitrogen) following the manufacturers protocol. 4 hours post transfection, the medium was removed and replaced with DMEM containing fetal bovine serum.

### Cell lysis

Cells were lysed in radioimmunoprecipitation (RIPA) buffer, containing 10 mM sodium phosphate (pH 7.4), 150 mM NaCl, 0.1% (w/v) SDS, 1% (v/v) Triton X100, 0.25% (w/v) deoxycholate, 5 mM EDTA, 25 mM NaF, 25 mM 2-glycerolphosphate, 200 μM Na_3_VO_4_ and protease inhibitors.

### Biotinylated LIMK1 peptide assay

FLAG-tagged ROCK2 was immunoprecipitated from transfected cell lysates with M2 monoclonal antibody conjugated to agarose (Sigma–Aldrich). Immunoprecipitates were washed three times in RIPA buffer, followed by an additional three times in activation buffer containing (50 μM Tris–HCl, 0.1 mM EGTA, 0.1% 2-mercaptoethanol), and finally resuspended in 30 μl of activation buffer containing various concentrations of ATP or N_6_(Benzyl)ATP. Biotinylated (N-terminal) LIMK1 peptide (DRKKRYTVVGNPY) was used as a substrate (200 μM). Samples were incubated for 30 min at 30°C, the reaction was terminated by the addition of 7.5 mM EDTA. The samples were then loaded onto streptavidin plates (Pierce) and bound for 2 hours at room temperature (24°C). The plates were then probed with phospho-LIMK (Cell Signaling) overnight at room temperature. Secondary decoration with IRDye® 680 anti-rabbit (Li-Cor) was performed at room temperature for 1.5 hours. Proteins were visualized by direct fluorescence scanning with a Li-Cor Odyssey Imager.

### Cellular homogenate labeling

3 × 10^6^ HEK293 cells were lysed in 2 ml hypotonic lysis buffer (20 mM Hepes, pH 7.4, 2 mM MgCl2, 200 μM Na3VO4, 0.25% NP-40, and protease inhibitors). Insoluble particulates were removed by centrifugation and 10% glycerol was added to the supernatant and stored at −80°C. Prior to *in vitro* labeling, the homogenate was heat inactivated for 10 min at 60°C. Cellular homogenate was then added to immunoprecipitated ROCK2 proteins with γ^32^P-N_6_(Benzyl)ATP in activation buffer. The reaction was incubated for 30 min at 30°C and terminated by the addition of 2X LDS sample buffer.

### Two-dimensional electrophoresis

Proteins were precipitated from samples with 4x the volume acetone, 10% TCA and 20 mM DTT at −20°C for 45 min. Proteins were then pelleted by centrifugation at 4°C for 15 min. The supernatant was removed and the protein pellet was subjected to an acetone wash, and then dried of residual acetone under vacuum for 2 min. The pellet was then resuspended in 260 μl 2D sample buffer (7 M Urea, 2 M Thio-Urea, 4% CHAPS, 2% DTT, 1% IPG buffer). To ensure protein solubilization, samples were incubated at room temperature for 15 min with intermittent vortexing. The sample was then transferred to a fresh Eppendorf tube and 250 μl was pipetted into a 13 cm strip holder (GE Healthcare). An immobiline dry strip (GE Healthcare) was then placed in the holder, overlayed with drystrip cover fluid. Both pH 3–11 NL and pH 4–7 L immobiline dry strips were used, see figures for details.

Proteins were then separated in the first dimension with the following isoelectric focusing (IEF) conditions: 10 hours rehydration, 5 hours 30 V, 1 hour 500 V, 1 hour 1000 V, 2.5 hour 8000 V, and 30 min 8000 V for ph3-11 NL. The conditions for pH 4–7 were as follows: 10 hours rehydration, 5 hours 30 V, 1 hour 500 V, 1 hour 1000 V, 2.5 hours 8000 V, and 0.55 hours 8000 V. Following IEF, strips were removed from the holders and pre-equilibrated in SDS-equilibration buffer (50 mM Tris–HCl, pH 8.8, 6 M Urea, 30% glycerol, 2% SDS, and 10 mg/ml DTT) for 1 hour at room temperature (24°C) with rocking. Proteins were then separated in the second dimension with 8% SDS-PAGE. After electrophoresis, gels were rinsed in ultrapure water and fixed in 50% ethanol, 10% acetic acid and stained using the Silver Quest kit (Invitrogen). ^32^P-labeled proteins identified by autoradiography were excised from the gel with a scalpel. Gel fragments were resuspended in 1% acetic acid in microcentrifuge tubes prior to analysis by mass spectrometry.

### EF1α1 *in vitro* kinase assay

FLAG-ROCK2 was immunoprecipitated from cell lysates with 20 μl of M2 FLAG monoclonal antibody conjugated to agarose (Sigma-Aldrich). Immunoprecipitates were washed three times with RIPA buffer, followed by two additional washes with kinase assay buffer containing 20 mM Tris pH 7.5, 50 mM NaCl, 5 mM MgCl2, 1 mM dithiothreitol (DTT), and finally resuspended in 30 μl of kinase assay buffer containing 10 μM ATP and 1 mCi [^32^P]ATP. Purified EF1α1 proteins were used as substrate. Samples were incubated for 30 min at 30°C, and terminated by the addition of 2x LDS sample buffer, and resolved on 4-12% Criterion™XT precast gel (Bio-Rad Laboratories). ^32^P-EF1α1 was visualized by phosphoimager (Bio-Rad Laboratories).

## Competing interest

The authors declare that they have no competing interests.

## Authors’ contributions

ALC contributed to the design of the study, performed the experiments, interpreted the results, and wrote the initial draft of the manuscript. RMG contributed to the generation of reagents for the study. MPS conceived and contributed to the design of the study, interpreted the results, and edited the final draft of the manuscript. All authors read and approved the final manuscript.
